# Effects of intestinal microbes on rheumatic diseases: A bibliometric analysis

**DOI:** 10.3389/fmicb.2022.1074003

**Published:** 2023-01-09

**Authors:** Runzhi Huang, Mengyi Zhang, Yuwei Lu, Dayuan Xu, Yifan Liu, Minghao Jin, Shuyuan Xian, Siqiao Wang, Xirui Tong, Jianyu Lu, Wei Zhang, Weijin Qian, Jieling Tang, Yiting Yang, Bingnan Lu, Zhengyan Chang, Xin Liu, Shizhao Ji

**Affiliations:** ^1^Department of Burn Surgery, The First Affiliated Hospital of Naval Medical University, Shanghai, China; ^2^Research Unit of Key Techniques for Treatment of Burns and Combined Burns and Trauma Injury, Chinese Academy of Medical Sciences, Shanghai, China; ^3^School of Medicine, Shanghai Jiao Tong University, Shanghai, China; ^4^School of Medicine, Tongji University, Shanghai, China; ^5^Department of Pathology, School of Medicine, Shanghai Tenth People’s Hospital, Tongji University, Shanghai, China; ^6^Department of Rheumatology and Immunology, Second Affiliated Hospital of Naval Medical University, Shanghai, China

**Keywords:** rheumatic diseases, intestinal microbiota, bibliometric analysis, host microbial interactions, dysbiosis

## Abstract

**Background:**

Rheumatic diseases (RD) are a group of multi-system inflammatory autoimmune diseases whose causes are still under study. In the past few decades, researchers have found traces of the association between rheumatic diseases and intestinal microbiota, which can partially explain the pathogenesis of rheumatic diseases. We aimed to describe the research trend and main divisions on how gut flora interreacts with rheumatic diseases, and discussed about the possible clinical applications.

**Methods:**

We analyzed bibliometric data from the Web of Science core collection (dated 15th May 2022). Biblioshiny R language software packages (bibliometrix) were used to obtain the annual publication and citations, core sources according to Bradford’s law, and country collaboration map. We designed and verified the keyword co-occurrence network and strategic diagram with the help of VOSviewer and CiteSpace, subdivided the research topic into several themes and identified research dimensions. The tables of most local cited documents and core sources were processed manually. Furthermore, the Altmetric Attention Score and the annual Altmetric Top 100 were applied to analyze the annual publication and citation.

**Results:**

From a total of 541 documents, we found that the overall trend of annual publication and citation is increasing. The major research method is to compare the intestinal microbial composition of patients with certain rheumatic disease and that of the control group to determine microbial alterations related to the disease’s occurrence and development. According to Bradford’s law, the core sources are *Arthritis and Rheumatology, Annals of the Rheumatic Diseases, Current Opinion in Rheumatology, Nutrients, Rheumatology, and Journal of Rheumatology.* Since 1976, 101 countries or regions have participated in studies of rheumatology and intestinal microbes. The United States ranks at the top and has the broadest academic association with other countries. Five themes were identified, including the pivotal role of inflammation caused by intestinal bacteria in the rheumatic pathogenesis, the close relationship between rheumatic diseases and inflammatory bowel disease, immunoregulation mechanism as a mediator of the interaction between rheumatic diseases and gut flora, dysbiosis and decreased diversity in intestine of patients with rheumatic diseases, and the influence of oral flora on rheumatic diseases. Additionally, four research dimensions were identified, including pathology, treatment, disease, and experiments.

**Conclusion:**

Studies on rheumatic diseases and the intestinal microbiota are growing. Attention should be paid to the mechanism of their interaction, such as the microbe-immune-RD crosstalk. Hopefully, the research achievements can be applied to diseases’ prevention, diagnosis, and treatment, and our work can contribute to the readers’ future research.

## 1 Introduction

Rheumatic diseases (RD) are a group of multi-system inflammatory autoimmune diseases with unknown causes. Its pathological lesions involve joints and the tissues around, mainly affecting small joints such as those of the hands and feet. Patients with early rheumatic diseases often have symptoms such as joint pain, swelling, and dysfunction. In advanced stages, patients may face joint stiffness and deformity, muscle atrophy, and even develop disability.

As chronic inflammatory diseases with long courses, severe damage and a high disability rate, rheumatic diseases seriously threaten patients’ health and bring great burden to their families and society. Take rheumatoid arthritis (RA) for example, according to the Global Burden of Disease 2010 Study, the global prevalence of RA was 0.24% (Cross et al., 2014). In the United States, estimates of RA prevalence tended to be higher, typically between 0.5% and 1% (Myasoedova et al., 2010; Hunter et al., 2017). Women are more likely to suffer from inflammatory autoimmune rheumatic diseases. It is estimated that one in 12 women will develop a rheumatic disease during their lifetime (Crowson et al., 2011). Additionally, patients may have some adverse long-term outcomes, such as physical disability (Guevara-Pacheco et al., 2017), work incapacity (Xiang et al., 2020), decreased life quality (Matcham et al., 2014), and even premature death (Bournia et al., 2021). A study shows that the disability score deteriorated by 1.8% per year in the Swiss national RA cohort (Heinimann et al., 2018).

The pathogenesis of rheumatic diseases involves multiple factors such as environment, inheritance, and immunity dysregulation, which is complicated and still not well-illustrated. As for treatment, non-steroidal anti-inflammatory drugs (NSAIDs), glucocorticoids, and disease-modifying antirheumatic drugs (DMARDs) are often required. However, multiple side effects are hard to avoid. NSAIDs may cause cardiovascular events, such as myocardial ischemia and stroke (Bhala et al., 2013). Glucocorticoid treatment can lead to osteoporosis and increase the risk of fracture (Rossini et al., 2017). The most common adverse reactions of DMARDs are gastrointestinal toxicity (such as nausea, vomiting, and diarrhea), hepatotoxicity, pulmonary toxicity, and so on (Wang et al., 2018). Therefore, mechanism research and drug discovery are of great urgency. Scientists are exploring unknown pathogenic mechanisms in the hope of finding new drug targets.

Microbes coexist with their human hosts all through their lives, acting as a crucial factor in human health and homeostasis. Among all parts of the human body, the gastrointestinal tract contains the largest proportion of commensal bacteria (Simon and Gorbach, 1984). There are over 1000 species of intestinal bacteria and at least 160 species in each individual (Qin et al., 2010). The microbiota in intestine is relatively stable all through one’s life (Reid et al., 2011), and was proved to have profound effects on the host’s local and systemic immune system (Honda and Littman, 2016). Therefore, maintenance of a balanced symbiosis is indispensable in preventing autoimmune rheumatic diseases.

Over the past few decades, numerous studies have linked microbiomes to various diseases, including obesity (Torres-Fuentes et al., 2017), diabetes (Gurung et al., 2020), asthma (Barcik et al., 2020), atherosclerosis (Jonsson and Bäckhed, 2017), inflammatory bowel disease (IBD) (Nishida et al., 2018), and many others related to the immune system. Among rheumatic diseases, rheumatoid arthritis (RA) (Maeda and Takeda, 2017), ankylosing spondylitis (AS) (Guggino et al., 2021), systemic lupus erythematosus (SLE) (Luo et al., 2018), psoriatic arthritis (PsA) (Myers et al., 2019), gout (Chu et al., 2021), scleroderma (Volkmann, 2017), and Behcet’s disease (Ye et al., 2018) have been confirmed to have connections with the microbiome. For example, *Prevotella* species are involved in the pathogenesis of RA. It was found that *Prevotella histicola* in intestinal microbiota suppressed the development of arthritis (Maeda and Takeda, 2017). Besides, SLE patients possessed an alteration in gut microbiota, including greater abundance of the bacterial phylum Proteobacteria and lower abundance in bacterial genera *Odoribacter* and *Blautia* (Luo et al., 2018). Consequently, it’s necessary for us to sort and summarize the currently known relationship between rheumatic disorders and intestinal microbes. It is of great importance to elucidate the pathogenesis of RD and it may provide new ideas for treatment in the future.

In recent years, many scholars have applied bibliometric analysis to the development of knowledge in health. The term “bibliometrics” was originally invented by Pritchard (1969), as “the application of mathematical and statistical methods to books and other media of communication”. It was created to meet the need for quantitative studies of scientific publications. From then on, bibliometrics has gradually evolved and was technically perfected. Since the 1980s, more and more researchers applied bibliometrics in the field of medicine and health care. Nowadays, application of bibliometrics in medical topics has translated into everyday clinical activities, which enables us to understand the evolution and main development directions of certain medical subject through scientific approach and provides guidance for further research (Kokol et al., 2021). Similarly, bibliometric analysis can be applied to explore the relationship between gut flora and rheumatic diseases. So far, there is no bibliometric study on the association between rheumatic diseases and intestinal microbiota. Therefore, we collected the related articles and analyzed them with the help of bibliometric analysis, with the purpose of identifying the distribution structure, quantity relations, and major research trends in the field. We hope our work will serve as a guide for identifying key knowledge and research priorities.

## 2 Materials and methods

### 2.1 Data sources and retrieval strategies

Data source was obtained from the Web of Science on 15th May 2022. Web of Science is the world’s largest and most comprehensive collection of academic resources, which contains more than 21,000 authoritative, high-impact journals and more than 300,000 academic conferences around the world, covering the fields of natural sciences, engineering technology, biomedicine, social sciences, arts, and humanities.

The retrieval strategy was as follows: [(TS = rheumatology) OR (TS = rheumatic disease) OR (TS = rheumatism)] AND [(TS = gut) OR (TS = intestin*) OR (TS = gastrointestin*) OR (TS = gastro-intestin*)] AND [(TS = microbiot*) OR (TS = microbiome*) OR (TS = flora) OR (TS = microflora) OR (TS = bacteria) OR (TS = prebiotic) OR (TS = probiotic)], language = English. Search in: All Databases. Collections: Web of Science Core Collection. A total of 541 documents were retrieved, all of which were open-access publications, including 253 articles, 161 reviews, 103 meeting abstracts, 17 editorial materials, and 7 other types of documents. All records were downloaded in a TXT format.

### 2.2 Data analysis

We analyzed the bibliographic data through several bibliometric analysis applications. Biblioshiny R language software packages were used to analyze and visualize article and journal performance, country influence and collaboration, research trends, and keywords. Biblioshiny, developed by Massimo Aria and Corrado Cuccurullo from the University of Naples and the University of Campania “Luigi Vanvitelli” (Italy), is powered by Bibliometrix and programmed in the R language, providing an intuitive and well-structured interface (Xiang et al., 2020). To analyze the annual publication and citation, Altmetric Attention Score (AAS) was applied. AAS is a weighted count of all the attention a research article receives online, which can be used for measuring a study’s influence in a comprehensive way, including social, academic, and other aspects. AAS and the list of annual Altmetric Top 100 was obtained from Altmetric.com website (Altmetric, London, United Kingdom). As regards to keyword analysis, with the help of VOSviewer (van Eck and Waltman, 2010) and CiteSpace (Chen, 2006), the co-occurrence network and the strategic diagram were performed, verified, and then optimized, both of which were constructed with top 250 keywords of high frequency selected by “keyword plus” with the algorithm “walktrap.” The tables of most local cited documents and core sources information were designed manually. Additionally, for a better understanding of the research trends and hotspots, we carefully read the related documents, subdivided the research topic into several themes, identified research dimensions, and discussed their future directions respectively.

## 3 Results

### 3.1 Annual publication and citation

**FIGURE 1:**
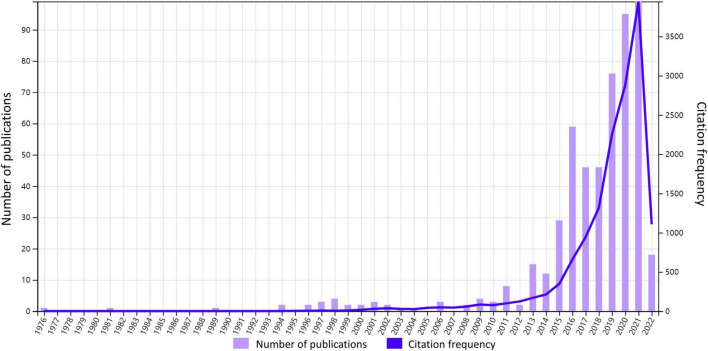
Annual citation and publication. The number of annual publications and frequency of citations from 1976 to 2022 are shown in the figure.

Annual publication and citation offer us a view of the general situation and development tendency of the field. As shown in Figure 1, from 1976 to 2022, a total of 541 documents have received 13762 citations. Despite the slight fluctuations in the number of annual publications, the overall trend is growing. We can approximately divide the development into three stages. 1976–1995 is an exploration period with sporadic production where only five documents were published and nearly zero was cited. 1996–2012 is a sprouting period. The number of annual publications and citations remained low while there were documents published and cited almost every year. The next period 2013–2021 is a period of expansion, which witnessed a rapid increase in annual publications, reaching a maximum of 97. At the same time, annual citations grew at an exponential rate.

In order to interpret such rapid growth from 2013 to 2021, we have explored the Altmetric Top 100 over the years, which includes the top 100 studies with the highest AAS of the year. It is found that from 2013 to 2015, there was one article each year discussing gut microbiota and reaching the annual Altmetric Top 100, the titles of which are “Intestinal microbiota metabolism of l-carnitine, a nutrient in red meat, promotes atherosclerosis” (Koeth et al., 2013), “Artificial sweeteners induce glucose intolerance by altering the gut microbiota (Suez et al., 2014),” and “Dietary emulsifiers impact the mouse gut microbiota promoting colitis and metabolic syndrome (Chassaing et al., 2015).” Their AASs are 1089, 4794, and 1835, while their annual ranks are 64, 3, and 57 respectively (Supplementary Figure S1). It follows that the intestinal flora was possibly a popular topic in 2013–2015. Therefore, we considered that the increased attention to gut microbiota research in the 2010s could be the reason for the growth spurt in annual publication during 2013–2021. What’s more, as we can see in Table 1, all of the top 20 most cited documents were released during this flourishing period. Especially the top 1 article achieves an AAS of 252 (Supplementary Figure S1) and ranks in the top 5% of all research outputs ever tracked by Altmetric, indicating its relatively high online attention, which we will further explain in the next chapter.

**TABLE 1:** Top 20 most local cited documents.

Rank	Title	Author	Article type	DOI	Year
1	The oral and gut microbiomes are perturbed in rheumatoid arthritis and partly normalized after treatment	Zhang et al., 2015	Article	doi: 10.1038/nm.3914	2015
2	Decreased bacterial diversity characterizes the altered gut microbiota in patients with psoriatic arthritis, resembling dysbiosis in inflammatory bowel disease	Scher et al., 2015	Article	doi: 10.1002/art.38892	2015
3	Dysbiosis contributes to arthritis development via activation of autoreactive t cells in the intestine	Maeda et al., 2016	Article	doi: 10.1002/art.39783	2016
4	An expansion of rare lineage intestinal microbes characterizes rheumatoid arthritis	Chen et al., 2016	Article	doi: 10.1186/s13073-016-0299-7	2016
5	Intestinal dysbiosis associated with systemic lupus erythematosus	Hevia et al., 2014	Article	doi: 10.1128/mBio.01548-14	2014
6	Analysis of fecal lactobacillus community structure in patients with early rheumatoid arthritis	Liu et al., 2013	Article	doi: 10.1007/s00284-013-0338-1	2013
7	Fecal microbiota study reveals specific dysbiosis in spondyloarthritis	Breban et al., 2017	Article	doi: 10.1136/annrheumdis-2016-211064	2017
8	Evidence of the immune relevance of *Prevotella copri*, a gut microbe, in patients with rheumatoid arthritis	Pianta et al., 2017	Article	doi: 10.1002/art.40003	2017
9	Alterations of the gut microbiome in Chinese patients with systemic lupus erythematosus	He et al., 2016	Article	doi: 10.1186/s13099-016-0146-9	2016
10	Review: microbiome in inflammatory arthritis and human rheumatic diseases	Scher et al., 2016	Review	doi: 10.1002/art.39259	2016
11	Intestinal dysbiosis and rheumatoid arthritis: a link between gut microbiota and the pathogenesis of rheumatoid arthritis	Horta-Baas et al., 2017	Review	doi: 10.1155/2017/4835189	2017
12	Microbiome and mucosal inflammation as extra-articular triggers for rheumatoid arthritis and autoimmunity	Brusca et al., 2014	Review	doi: 10.1097/BOR.0000000000000008	2014
13	Dysbiosis and zonulin upregulation alter gut epithelial and vascular barriers in patients with ankylosing spondylitis	Ciccia et al., 2017	Article	doi: 10.1136/annrheumdis-2016-210000	2017
14	Quantitative metagenomics reveals unique gut microbiome biomarkers in ankylosing spondylitis	Wen et al., 2017	Article	doi: 10.1186/s13059-017-1271-6	2017
15	Th17 responses and natural IGM antibodies are related to gut microbiota composition in systemic lupus erythematosus patients	López et al., 2016	Article	doi: 10.1038/srep24072	2016
16	Gut microbiota in human systemic lupus erythematosus and a mouse model of lupus	Luo et al., 2018	Article	doi: 10.1128/AEM.02288-17	2018
17	How the microbiota shapes rheumatic diseases	Van de Wiele et al., 2016	Review	doi: 10.1038/nrrheum.2016.85	2016
18	Lupus nephritis is linked to disease-activity associated expansions and immunity to a gut commensal	Azzouz et al., 2019	Article	doi: 10.1136/annrheumdis-2018-214856	2019
19	The intestinal microbiome in spondyloarthritis	Gill et al., 2015	Review	doi: 10.1097/BOR.0000000000000187	2015
20	Association of systemic sclerosis with a unique colonic microbial consortium	Volkmann et al., 2016	Article	doi: 10.1002/art.39572	2016

Local cited documents refer to the documents cited by other articles in our database.

### 3.2 Most cited documents

In Table 1, we present the top 20 most local cited documents, among which there are 15 articles and 5 reviews.

The content of the articles was analyzed. The major research method is to compare the microbial composition of patients with certain rheumatic disease and that of the healthy controls to determine whether certain microbial genus alterations are associated with the occurrence and development of the disease. The most studied disease is rheumatoid arthritis, while other rheumatic diseases, including systemic lupus erythematosus, ankylosing spondylitis, psoriatic arthritis, and systemic sclerosis, were also researched. Most of these studies have found that patients with rheumatic disease exhibit decreased diversity in gut flora and a relative increase or decrease in abundance of specific species. In addition to the analysis of intestinal microbes, dental and salivary samples were also examined, where similar changes were observed. As illustrated in the most cited article, dysbiosis (altered microbial composition) was detected in fecal, dental, and salivary samples from patients with RA, with a decrease in *Haemophilus* spp. as well as an increase in *Lactobacillus salivarius* existing at all three sites compared with healthy controls (Zhang et al., 2015). What’s more, there is a study pointing out that the degree of these bacterial changes was correlated with disease duration and autoantibody levels (Chen et al., 2016). From these results, it can be inferred that detecting microbe alterations can be used in screening and diagnosis of multiple rheumatic diseases, and treating the dysbiosis might be a way to improve diseases.

The remaining five documents are reviews mainly discussing the role and influence of intestinal dysbiosis on the development of various rheumatic diseases. The presence of bacteria in mucosal surfaces, such as the intestine, the gingiva, and the respiratory tree, is able to trigger host immune responses and cause inflammation (Brusca et al., 2014). In the review written by Gill et al. (2015), it’s also pointed out that, except for rheumatic diseases, various inflammatory disorders, such as IBD, are affected by microbiota.

As mentioned above, the most local and global cited document is a metagenome-wide association study (MGWAS) published by a research team, led by Zhang et al. (2015), of Peking Union Medical College Hospital. This article is the most well-known one which utilized novel sequencing technologies into intestinal dysbiosis of rheumatic diseases. Through metagenomic shotgun sequencing, microbial alterations in mouth, gut and saliva of patients with RA were identified, and since then, a growing number of studies have used similar approached to explore the field. With the development of sequencing technology, compositional and functional changes of gut flora have been found in a variety of rheumatic diseases, such as AS (Wen et al., 2017) and SLE (Tomofuji et al., 2021). In this way, dysbiosis of specific rheumatic diseases can be understand more precisely, so that targeted therapies can be applied.

### 3.3 Source analysis

**FIGURE 2:**
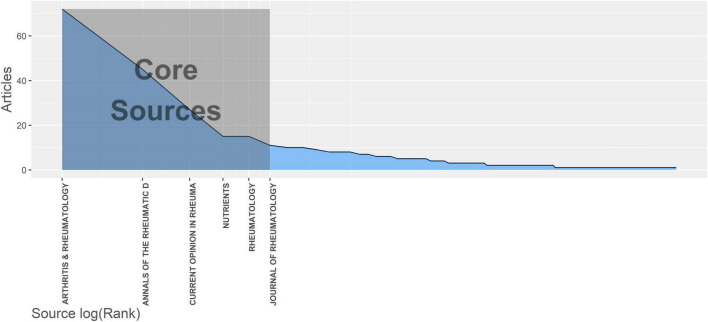
Core sources according to Bradford’s law. According to Bradford’s law, one third of the articles in the field lie in the zone of core sources, which were published by six different sources.

All selected articles were published in one of 199 sources. Six journals have published more than 10 articles, accounting for 34.2% (185 of 541) of the total articles. We applied Bradford’s law to identify core sources of the research on intestinal microbes and rheumatic diseases (Bradford, 1934). As depicted in Figure 2, the core sources are *Arthritis and Rheumatology, Annals of the Rheumatic Diseases, Current Opinion in Rheumatology, Nutrients, Rheumatology and Journal of Rheumatology*, among which four journals were classified as Q1 and the rest two were classified as Q2 by the Journal Citation Reports standard in 2020. Five journals were in the JCR category of rheumatology and five had an impact factor greater than five (Table 2).

**TABLE 2:** Core sources information.

Rank	Journal	Number of publications	Proportion	Journal impact factor (2020)	Category quartile (2020)	JCR category	Category rank
1	Arthritis and Rheumatology	72	13.3%	10.995	Q1	RHEUMATOLOGY	3/34
2	Annals of the Rheumatic Diseases	45	8.3%	19.103	Q1	RHEUMATOLOGY	2/34
3	Current Opinion in Rheumatology	27	5.0%	5.006	Q2	RHEUMATOLOGY	11/34
4	Nutrients	15	2.8%	5.719	Q1	NUTRITION and DIETETICS	17/88
5	Rheumatology	15	2.8%	7.580	Q1	RHEUMATOLOGY	5/34
6	Journal of Rheumatology	11	2.0%	4.666	Q2	RHEUMATOLOGY	14/34

Data sources: Journal Citation Reports™ 2020.

### 3.4 Country influence and collaboration

Since 1976, 53 countries or regions have participated in studies of rheumatology and intestinal microbes. The United States tops the ranking of country production with a frequency (number of documents) of 527, followed by China (330), Italy (191), the United Kingdom (116), and Japan (111). Correspondingly, the top five most cited countries are the same, including the United States (4043), China (2175), the United Kingdom (1136), Italy (800), and Japan (760). The more citations a country has, the stronger its scientific influence is in this field. Therefore, it reveals that these five countries are the most active, prolific, and influential in this field.

**FIGURE 3:**
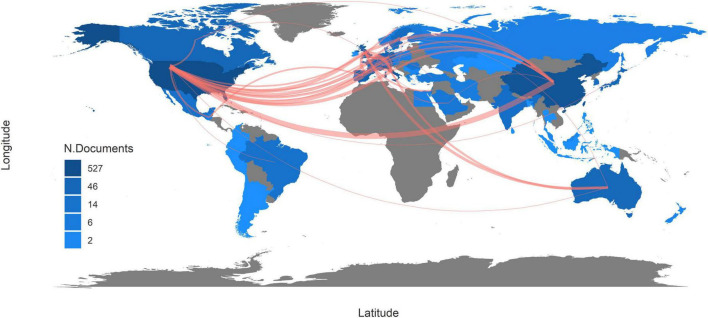
Country collaboration map. The red line indicates that there was collaboration between two countries. The more connections two countries have, the thicker the line is. The deeper the color of a country is, the more documents are published in this country.

Along with the country collaboration map (Figure 3), a complete picture of academic performance and country collaboration is presented. The figure was created based on the frequency of cooperation between two countries. If there exists research cooperation between two countries, those two areas will be connected by a line on the map. The more connections two countries have, the thicker the line is. The United States has the broadest academic associations with other countries or regions while its collaboration with China is the tightest. The United Kingdom and Australia also have strong academic partnerships. In contrast, communication among other countries could be strengthened. With more international cooperative research projects, the disturbance of local lifestyle habits and regional features on microbial characteristics will be eliminated, and the research findings can be more accurate and be more widely applicated.

### 3.5 Keyword analysis

#### 3.5.1 Keyword co-occurrence network

**FIGURE 4:**
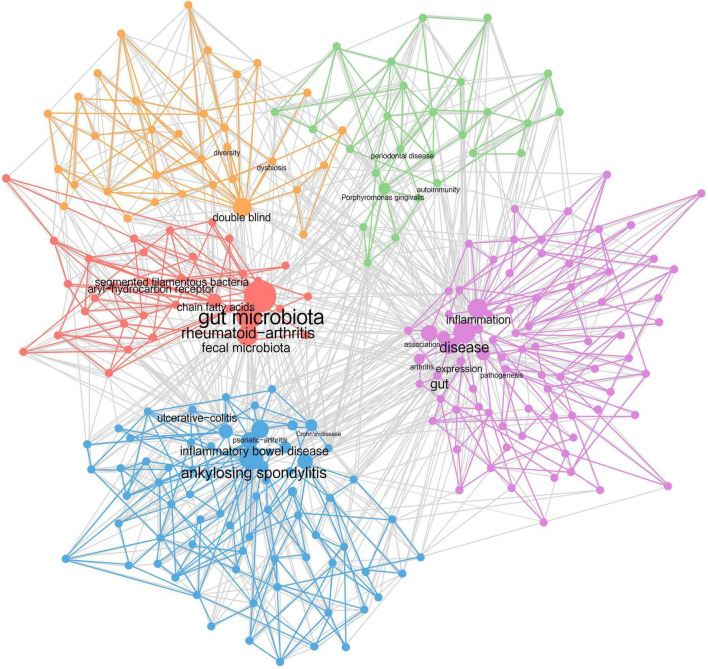
Keyword co-occurrence network. Each dot represents a keyword. The higher the frequency it has, the bigger the dot is. Keywords showing up in the same document are wired together, forming five clusters of dots of different colors.

Keywords provide an overview of the research content in a highly refined and generalized way. Through connecting the keywords existing in the same article, a keyword co-occurrence network was formed (Figure 4), allowing us to further analyze the research topics and trends. The top 10 most frequently occurring keywords are gut microbiota (466), ankylosing-spondylitis (346), rheumatoid-arthritis (321), disease (272), gut (263), inflammatory bowel disease (252), t-cells (211), association (191), arthritis (188), and Crohn’s disease (186). Each keyword is indicated by a node, the size of which shows its occurrence time. Based on this, keywords with close relations were grouped into one cluster, representing an aspect of the core research field, and nodes with the same color belong to the same cluster. In our analysis, five clusters were recognized.

Cluster 1 (purple): Intestinal bacteria lead to rheumatic diseases through joints’ inflammation. Keywords include inflammation, arthritis, pathogenesis, association, etc.
Cluster 2 (blue): Rheumatic diseases are closely associated with inflammatory bowel disease. Keywords include ankylosing spondylitis, inflammatory bowel disease, psoriatic arthritis, Crohn’s disease, ulcerative colitis, etc.
Cluster 3 (red): Rheumatic diseases and intestinal microbiota are interrelated by immunoregulation. Keywords include gut microbiota, rheumatoid arthritis, chain fatty acids, segmented filamentous bacteria, aryl-hydrocarbon receptor, etc.
Cluster 4 (orange): Intestinal dysbiosis and loss of microbial diversity exist in patients with rheumatic diseases. Keywords include dysbiosis, diversity, double blind, etc.
Cluster 5 (green): The occurrence of rheumatic diseases is also related to the oral flora. Keywords include *Porphyromonas gingivalis*, periodontal disease, autoimmunity, etc.

Furthermore, four research dimensions were identified, including pathology, treatment, disease, and experiments. The pathology part was divided into macro and micro level. The themes of clusters and the research dimensions were cross-tabulated, resulting in a taxonomy presented in Supplementary Table S2.

#### 3.5.2 Strategic diagram

Based on the keyword co-occurrence network, the centrality and density of each cluster were calculated and a strategic diagram was drawn, with each circle representing a cluster of the same color. Centrality indicates the degree of closeness of one topic with the others, while density indicates the grade of maturity of one theme. Through the strategic diagram, we can further understand the development situation and the future direction of each topic.

**FIGURE 5:**
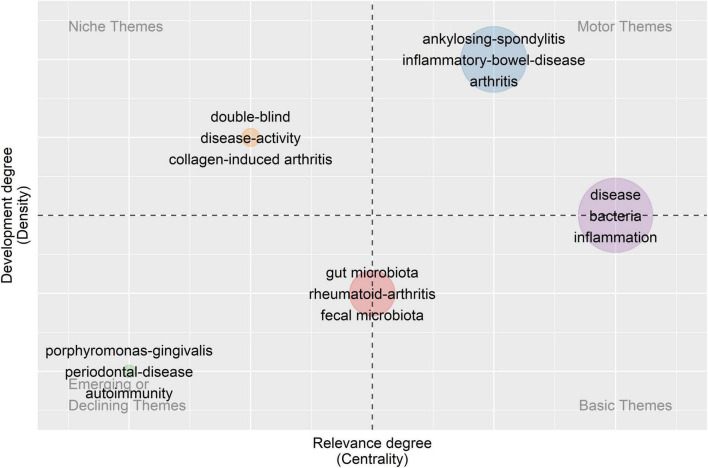
Strategic diagram. Five clusters of keywords are distributed in a coordinate system according to the centrality and density of each cluster. Centrality indicates the degree of closeness of one topic with the others while density means the grade of maturity of one theme.

As shown in Figure 5, there are two clusters located on the axis. The mechanism of interaction between intestinal flora and rheumatic diseases (circle red) achieves a low density, which means it is still underdeveloped and requires further research. For example, it is still under study how gut microbiota contribute to the pathogenesis of rheumatic disorders. The topic of intestinal-bacteria-caused inflammation in rheumatic diseases (circle purple) is of high centrality, which means it connects closely with other subjects, indicating its role as the basis of all relevant research. Inflammation triggered by intestinal bacteria is thought to be a possible pathological basis for rheumatoid disorders (Toivanen, 2003; Maeda et al., 2016). As for the other three clusters distributed in different quadrants, the association between RD and IBD (circle blue) lies in the first quadrant with high centrality and density. As the core topic in this field, its research system has been formed and closed to completion. It is confirmed by a range of research that rheumatoid disorders, such as AS and SLE, could happen to one patient simultaneously or successively with IBD (Shor et al., 2016; Klingberg et al., 2017). In the second quadrant, dysbiosis and low bacterial diversity in patients with rheumatic disease (circle orange) is a relatively developed theme isolated from the other topics, achieving relatively low centrality and high density. The changes in gut flora are always observed in patients with rheumatoid disorders, but their cause remains to be further discussed. In the third quadrant, both the centrality and density of the cluster are low, indicating that the topic is disappearing. Shared inflammatory mechanism within RA and periodontal disease (circle green) was first stated by Snyderman and McCarty (1982) while the hypothesis referring to *P. gingivalis* was first published by Rosenstein et al. (2004). Subsequently, research on this topic sprang up in the 2000s and 2010s, and the mechanism of PD’s effects on RA was explored at large. In the past decade, new discoveries have rarely been reported. Meanwhile, *P. gingivalis* has been the only oral bacterium associated with rheumatic diseases so far.

## 4 Discussion

Deducing from the above analysis, it is certain that rheumatic diseases are closely related to changes in gut flora while particular changes in bacterium genera occur in specific rheumatic diseases. However, it is still unclear how intestinal flora affects the occurrence and progression of RD, which needs to be further probed before it can be used in clinical treatments.

By observing the keyword co-occurrence network carefully and reading the relevant articles, as inferred from cluster 1, intestinal bacteria can cause inflammation in joints, resulting in rheumatic diseases. Germ-free SKG mice transplanted with fecal bacteria from RA patients and induced with zymosan would develop severe arthritis, with an increase in the number of CD4 + T cells and IL-17-producing T helper (Th17) cells in the intestine (Maeda et al., 2016). Another experiment demonstrated that infections with intestinal pathogens such as *Yersinia, Salmonella*, or *Shigella* may trigger reactive arthritis, as the catabolite of these pathogens can be transferred into the synovial tissues, generating local inflammation (Toivanen, 2003). Therefore, intestinal flora and its inflammation play an important role in the pathogenesis of rheumatic disease, while gut and joint inflammation are closely related pathologically, as can also be concluded from cluster 2.

In cluster 2, it is recognized that rheumatic diseases are closely associated with IBD, which includes ulcerative colitis (UC) and Crohn’s disease (CD). Spondyloarthritis (SpA) is a group of chronic inflammatory rheumatic diseases that includes ankylosing spondylitis, psoriatic arthritis, juvenile spondyloarthritis (JSpA), and enteroarthritis (EA), and it can be classified as axial or peripheral. According to the statistics, up to 40% of IBD patients show articular manifestations, while many patients with rheumatic disease are disturbed by intestinal inflammation at the same time (Klingberg et al., 2017). As reported, IBD and Spa have a common genetic predisposition and pathogenic mechanisms (Ashrafi et al., 2021). However, the exact mechanism of how the intestinal microbiome leads to the association between rheumatic disease and IBD is still under study. The hypothesis includes increased intestinal permeability, impairment in immune tolerance, and migration of intestinal microbial components and activated immune cells to the joint (Yeoh et al., 2013). Depletion of *Faecalibacterium prausnitzii* has been found in various inflammatory disorders, including SpA and IBD, which is most likely an important pathogenic factor (Gill et al., 2015). Additionally, Th17 cells have been implicated in the pathogenesis, resulting in chronic tissue inflammation (Gaffen et al., 2014).

As depicted in cluster 3, rheumatic diseases and intestinal microbiota are mainly interrelated by immunoregulation. When an inappropriate immune response occurs in chronic inflammation, microbial tolerance can be disrupted, resulting in the expansion or contraction of specific bacterial communities. In contrast, changes in gut microbiome composition can affect the immune system, leading to the occurrence of rheumatic diseases. This idea was supported by experiments on animal models, where HLA-B27 transgenic rats avoided arthritis in germ-free conditions, and inflammatory manifestations recurred after recolonized with certain bacteria (Taurog et al., 1999).

Microbe-immune-RD crosstalk is linked through short-chain fatty acid (SCFA) production and aryl hydrocarbon receptor (AhR) activation. SCFAs, including acetate, propionate, and butyrate, are the products of dietary fiber fermentation by gut bacteria (Koh et al., 2016), which serve as the link between the intestinal flora and the mucosal immune system (Arpaia and Rudensky, 2014; Zeng and Chi, 2015). It is reported that pentanoate acts as a regulator of immunometabolism by suppressing IL-17A production and inducing IL-10 expression in effector T cells (Luu et al., 2019). Additionally, SCFAs enhance intestinal barrier function by inducing regulatory T cells (Tregs) differentiation in the colon (Smith et al., 2013; Kelly et al., 2015). In patients with RA, through high-fiber dietary intervention to elevate SCFA content, circulating Tregs and the Th1/Th17 ratio increased, while the physical functioning and quality of life were improved significantly (Häger et al., 2019).

Aryl hydrocarbon receptor plays a critical role as a modulator of immune function to balance intestinal immune tolerance and activation (Esser and Rannug, 2015; Hubbard et al., 2015). The activity of AhR is a determinant of Th17 and Treg cell differentiation, whose balance profoundly influences immune response on the mucosal surface (Ehrlich et al., 2018). What’s more, AhR helps to ensure the tolerance of intraepithelial T lymphocytes and innate lymphoid cells (ILCs) by driving the expression of IL-22p in order to maintain epithelial barrier integrity and microbial homeostasis (Behnsen et al., 2014).

What’s more, there is a kind of intestinal bacteria reported to have a powerful immune-modulating function. Segmented Filamentous Bacteria (SFB) is a gram-positive *Clostridium* spp. colonizing the surface of epithelial cells in small intestine (Davis and Savage, 1974). It can stimulate Th17 cell differentiation and promote surface immunoglobulin A (sIgA) secretion in intestine, playing an important role in the induction of autoimmune diseases (Kumar et al., 2016). While germ-free mice rarely develop experimental autoimmune encephalomyelitis (EAE), those mono-colonized with SFB achieve CNS inflammation, and symptoms can be relieved with treatment of pentanoate (Luu et al., 2019). It is also found that in mice with Ahr−/−, the abundance of SFB increases significantly while an enhanced inflammatory state is displayed within the intestine (Murray et al., 2016).

Cluster 4 shows that dysbiosis and loss of microbial diversity both exist in the intestine of patients with rheumatic diseases. Disease-specific bacterial alterations and microbiota biodiversity restrictions were evidenced in spondyloarthritis. The number of observed species significantly decreased compared with healthy controls. Specifically, the abundance of *Ruminococcus gnavus* in SpA patients increased and positively correlated with SpA activity, which is consistent with the possible effect of *R. gnavus* on triggering or maintaining inflammatory status due to its mucolytic ability (Breban et al., 2017). Additionally, patients with SLE exhibited a decreased ratio of *Firmicutes*/*Bacteroidetes* while patients with RA showed an increase in *Prevotella copri* (Hevia et al., 2014; Pianta et al., 2017).

As we can see in cluster 5, the occurrence of rheumatic diseases is not only related to intestinal microbiomes but also related to the oral flora, whose diversity is second only to the gut (Dewhirst et al., 2010). Some specific strains can destroy the dynamic balance between oral flora and the host, causing autoimmune disorders and resulting in rheumatic diseases (Whitmore and Lamont, 2014). Periodontal disease (PD), for instance, is a common oral disease occurring in the periodontium, caused by a variety of bacterial infections (Kawar et al., 2011). Higher levels of disease activity has been found in patients with PD who later developed RA (Hashimoto et al., 2015). *Porphyromonas gingivalis*, a gram-negative anaerobic bacteria, is one of the important pathogenic bacterial species of PD (Wade, 2013). It has been confirmed to have a potential association with RA. Compared with healthy controls, RA patients had a higher titer of anti-*P. gingivalis* antibodies and increased antibody concentration preceded the onset of joint symptoms (Johansson et al., 2016; Kharlamova et al., 2016).

It is assumed that the existence of *P. gingivalis* contributes to the progression of RA by inducing the production of anticitrullinated protein antibodies (ACPA) (Wegner et al., 2010), a specific marker for the diagnosis and prognosis of RA (Barra et al., 2013; Okada et al., 2013). *P. gingivalis* is the sole microorganism reported that can produce peptidylarginine deiminase (PAD), an enzyme that can catalyze the conversion of arginine residue into citrulline residue, playing an important role in the formation of ACPA (Mangat et al., 2010). New epitopes can be created by the citrullination of proteins in the mucosa such as vimentin and keratin, causing the loss of immune tolerance and thus the presence of ACPA (Rosenstein et al., 2004). Citrullinated alpha-enolase peptide 1 (CEP-1), a major antigenic target of ACPA, shows sequence similarity and cross-reacts with enolase from *P. gingivalis* (Lundberg et al., 2008). Moreover, studies show that by controlling periodontal infection and treating PD, symptoms of RA began to subside (Erciyas et al., 2013), with a reduction in levels of TNF-α, ACPAs, and anti-*P. gingivalis* antibodies (Ortiz et al., 2009; Okada et al., 2013).

Variations on gut flora could be utilized in disease prevention, diagnosis, and treatment. The diagnosis of rheumatic diseases with bacteria seems to be prospective. In a study of bacterial changes in patients with gout, a diagnosis model was established based on 17 genera of intestinal microbiota sampled from stool, which showed promising diagnosis sensitivity. The accuracy of this microbiota-based predictive model was 81.7% when applied to the experiment and control groups, and it reached 88.9% in the validation group, which is higher than the diagnosis based on blood uric acid (71.3%) (Guo et al., 2016). Similarly, a diagnosis model for SLE based on five genera and two phyla of the oral microbiota achieved a possibility of 95.3% in distinguishing SLE patients from healthy controls (Li et al., 2020). Therefore, the microbiota-based predictive model may be a potential tool for screening and earlier diagnosis of rheumatic diseases, but it still requires verification in larger populations.

As the effects of intestinal microbiota on diseases have become better understood, dietary interventions and microbiota transplantation are being used to treat dysbiosis. As shown in Supplementary Table S2, some strains of the genera *Bifidobacterium* and *Lactobacillus* are commonly served as probiotic supplementation (Salazar et al., 2011), also known as live microorganisms that produce beneficial effects on hosts’ health when given in sufficient numbers (Food and Agriculture Organization of the United Nations, and World Health Organization, 2006). Several studies verified that administration of *Lactobacillus casei* for weeks improved symptoms and the pathophysiological index in RA patients (Alipour et al., 2014; Zamani et al., 2016), since patients with RA may have a decreased abundance of Lactobacillus spp., as shown in previous research (Davis and Savage, 1974). Nevertheless, more research needs to be conducted about the proper formulation and dose of the probiotics as well as the appropriate frequency and course of the treatment.

Prebiotics, defined as a group of food ingredients that benefit host health by regulating the microbiome, benefit our health by reducing oxidative stress, enhancing anti-inflammatory SCFA activity, and increasing IL-1Ra, IL-10, and IL-18 production (Peters et al., 2019). It is possible for prebiotics, such as oligosaccharides, dietary fiber, and resistant starch, to have a positive effect on treating rheumatic diseases. However, according to a number of reviews, the existing evidence is limited and inconclusive (Badsha, 2018; Macfarlane et al., 2018).

Postbiotic intake may be effective for treating rheumatic diseases. As represented by SCFA, postbiotics are soluble factors secreted by the metabolic activities of living bacteria or released components after bacterial death with beneficial effects on the host. Previous studies have shown that direct SCFA intake or a high-fiber diet can inhibit bone loss and suppress experimental arthritis (Lucas et al., 2018; Zaiss et al., 2019).

Additionally, fecal microbiota transplant (FMT) can potentially be a novel treatment for rheumatic diseases. By transferring stool from a healthy donor to a patient, dysbiosis is hoped to be controlled. This therapy was first used to treat *Clostridium difficile* infection and achieved a 90% success rate (Quraishi et al., 2017). As for rheumatic diseases, there exist a few trials of FMT ongoing, involving psoriatic arthritis and systemic sclerosis (Kragsnaes et al., 2018; Hoffmann-Vold et al., 2021). However, it is of great difficulty to predict the efficacy due to the unique intestinal microbiota of every individual donor, and the procedure needs to be further standardized as far as the donor status, sample preparation, dosage regimen, and therapeutic effect evaluation are concerned.

All the treatments mentioned above remain in their infancy in treating rheumatic diseases, but their potential is worth expectation, given the demand to try novel approaches, along with the growing understanding of interactions between microbes and human hosts. In the future, more bioinformatics methods can be applied to relevant researches, such as integrated regulatory network analysis of single-cell sequencing of individual bacterium and bulk sequencing of gut microflora. With the combination of genomics, transcriptomics, and proteomics studies, the pathogenesis of rheumatic diseases as well as the mechanism of gut flora’s influence on rheumatic diseases can be further explored.

As the first study that attempts to apply bibliometric analysis to the relationship between rheumatic diseases and gut microbiota, hopefully our study could bring new sights to our readers. By scientifically analyzing hundreds of papers, the findings are relatively more trustworthy. Researchers may learn about the historical evolution and future direction of the topic and find out a more suitable orientation for their further research. For students and those who are new to this field, by reading our article, they could have a general understanding of the interaction between rheumatic diseases and gut flora and might find out the sub-topic they want to explore in development. However, there still exist some limitations in our study. First, since our data was obtained solely from the Web of Science Core Collection, there are other relevant articles not included in our study. Second, by looking at the most cited documents, we can recognize the important articles and research directions in history, but it is of difficulty to identify the popular direction at present, since the citation amount of the paper published in the last few years cannot be competitive with those published decades ago. Nowadays, the popularity of one study also depends on social influence and exposure degree to the general public, except for citation number as a recognition degree in academia, which demands further analysis other than bibliometric analysis to assess.

## 5 Conclusion

In this article, we examined the performance of the documents, sources, and countries in the field. We focused on five branches and summarized knowledge on each aspect of the interaction between rheumatic diseases and intestinal microbiota from a visualization and bibliometric perspective. The research trends are still growing, and more attention should be paid to the mechanisms and pathogenesis, such as the microbe-immune-RD crosstalk. We hope our study can provide new knowledge and fresh thinking for readers, and hopefully more research achievements can be applied to clinical practice in the purpose of expanding the methods of disease prevention, diagnosis, and treatment, for the maximum benefit of RD patients.

## Data availability statement

Publicly available datasets were analyzed in this study. This data can be found here: Web of Science™ (WOS, http://www.webofknowledge.com).

## Author contributions

All authors did the conception and design, collected and/or assembled the data, carried out the data analysis and interpreted the data, wrote the manuscript, and finally approved the manuscript.

## References

[B1] Alipour B. Homayouni-Rad A. Vaghef-Mehrabany E. Sharif S. Vaghef-Mehrabany L. Asghari-Jafarabadi M. (2014). Effects of *Lactobacillus casei* supplementation on disease activity and inflammatory cytokines in rheumatoid arthritis patients: a randomized double-blind clinical trial. *Int. J. Rheum Dis.* 17 519–527. 10.1111/1756-185X.12333 24673738

[B2] Arpaia N. Rudensky A. (2014). Microbial metabolites control gut inflammatory responses. *Proc. Natl. Acad. Sci. U.S.A.* 111 2058–2059. 10.1073/pnas.1323183111 24434557 PMC3926042

[B3] Ashrafi M. Kuhn K. Weisman M. (2021). The arthritis connection to inflammatory bowel disease (IBD): why has it taken so long to understand it? *RMD Open* 7:e001558. 10.1136/rmdopen-2020-001558 33863841 PMC8055104

[B4] Azzouz D. Omarbekova A. Heguy A. Schwudke D. Gisch N. Rovin B. (2019). Lupus nephritis is linked to disease-activity associated expansions and immunity to a gut commensal. *Ann Rheum Dis.* 78 947–956. 10.1136/annrheumdis-2018-214856 30782585 PMC6585303

[B5] Badsha H. (2018). Role of diet in influencing rheumatoid arthritis disease activity. *Open Rheumatol. J.* 12 19–28. 10.2174/1874312901812010019 29515679 PMC5827298

[B6] Barcik W. Boutin R. Sokolowska M. Finlay B. (2020). The role of lung and gut microbiota in the pathology of asthma. *Immunity* 52 241–255. 10.1016/j.immuni.2020.01.007 32075727 PMC7128389

[B7] Barra L. Scinocca M. Saunders S. Bhayana R. Rohekar S. Racapé M. (2013). Anti-citrullinated protein antibodies in unaffected first-degree relatives of rheumatoid arthritis patients. *Arthritis Rheum.* 65 1439–1447. 10.1002/art.37911 23450693

[B8] Behnsen J. Jellbauer S. Wong C. Edwards R. George M. Ouyang W. (2014). The cytokine IL-22 promotes pathogen colonization by suppressing related commensal bacteria. *Immunity* 40 262–273. 10.1016/j.immuni.2014.01.003 24508234 PMC3964146

[B9] Bhala N. Emberson J. Merhi A. Abramson S. Arber N. Baron J. (2013). Vascular and upper gastrointestinal effects of non-steroidal anti-inflammatory drugs: meta-analyses of individual participant data from randomised trials. *Lancet* 382 769–779. 10.1016/S0140-6736(13)60900-9 23726390 PMC3778977

[B10] Bournia V. Fragoulis G. Mitrou P. Mathioudakis K. Tsolakidis A. Konstantonis G. (2021). All-cause mortality in systemic rheumatic diseases under treatment compared with the general population, 2015–2019. *RMD Open* 7:e001694. 10.1136/rmdopen-2021-001694 34728554 PMC8565571

[B11] Bradford S. (1934). Sources of information on specific subjects reprinted from engineering. *Illustrated Wkly. J.* 137 85–86.

[B12] Breban M. Tap J. Leboime A. Said-Nahal R. Langella P. Chiocchia G. (2017). Faecal microbiota study reveals specific dysbiosis in spondyloarthritis. *Ann. Rheum Dis.* 76 1614–1622. 10.1136/annrheumdis-2016-211064 28606969

[B13] Brusca S. Abramson S. Scher J. (2014). Microbiome and mucosal inflammation as extra-articular triggers for rheumatoid arthritis and autoimmunity. *Curr. Opin. Rheumatol.* 26 101–107. 10.1097/BOR.0000000000000008 24247114 PMC4011633

[B14] Chassaing B. Koren O. Goodrich J. Poole A. Srinivasan S. Ley R. (2015). Dietary emulsifiers impact the mouse gut microbiota promoting colitis and metabolic syndrome. *Nature* 519 92–96. 10.1038/nature14232 25731162 PMC4910713

[B15] Chen C. (2006). CiteSpace II: Detecting and visualizing emerging trends and transient patterns in scientific literature. *J. Am. Soc. Inf. Sci. Technol.* 57 359–377. 10.1002/asi.20317

[B16] Chen J. Wright K. Davis J. Jeraldo P. Marietta E. Murray J. (2016). An expansion of rare lineage intestinal microbes characterizes rheumatoid arthritis. *Genome Med.* 8:43. 10.1186/s13073-016-0299-7 27102666 PMC4840970

[B17] Chu Y. Sun S. Huang Y. Gao Q. Xie X. Wang P. (2021). Metagenomic analysis revealed the potential role of gut microbiome in gout. *NPJ Biofilms Microbiomes.* 7:66. 10.1038/s41522-021-00235-2 34373464 PMC8352958

[B18] Ciccia F. Guggino G. Rizzo A. Alessandro R. Luchetti M. Milling S. (2017). Dysbiosis and zonulin upregulation alter gut epithelial and vascular barriers in patients with ankylosing spondylitis. *Ann. Rheum. Dis.* 76 1123–1132. 10.1136/annrheumdis-2016-210000 28069576 PMC6599509

[B19] Cross M. Smith E. Hoy D. Carmona L. Wolfe F. Vos T. (2014). The global burden of rheumatoid arthritis: estimates from the global burden of disease 2010 study. *Ann Rheum Dis.* 73 1316–1322. 10.1136/annrheumdis-2013-204627 24550173

[B20] Crowson C. Matteson E. Myasoedova E. Michet C. Ernste F. Warrington K. (2011). The lifetime risk of adult-onset rheumatoid arthritis and other inflammatory autoimmune rheumatic diseases. *Arthritis Rheum.* 63 633–639. 10.1002/art.30155 21360492 PMC3078757

[B21] Davis C. Savage D. (1974). Habitat, succession, attachment, and morphology of segmented, filamentous microbes indigenous to the murine gastrointestinal tract. *Infect Immun.* 10 948–956. 10.1128/iai.10.4.948-956.1974 4426712 PMC423041

[B22] Dewhirst F. Chen T. Izard J. Paster B. Tanner A. Yu W. (2010). The Human Oral Microbiome. *J. Bacteriol.* 192 5002–5017. 10.1128/JB.00542-10 20656903 PMC2944498

[B23] Ehrlich A. Pennington J. Bisson W. Kolluri S. Kerkvliet N. (2018). TCDD, FICZ, and Other High Affinity AhR Ligands Dose-Dependently Determine the Fate of CD4+ T Cell Differentiation. *Toxicol. Sci.* 161 310–320. 10.1093/toxsci/kfx215 29040756 PMC5837604

[B24] Erciyas K. Sezer U. Ustün K. Pehlivan Y. Kisacik B. Senyurt S. (2013). Effects of periodontal therapy on disease activity and systemic inflammation in rheumatoid arthritis patients. *Oral Dis.* 19 394–400. 10.1111/odi.12017 22998534

[B25] Esser C. Rannug A. (2015). The aryl hydrocarbon receptor in barrier organ physiology, immunology, and toxicology. *Pharmacol. Rev.* 67 259–279. 10.1124/pr.114.009001 25657351

[B26] Food and Agriculture Organization of the United Nations World Health Organization (2006). Probiotics in food. Health and nutritional properties and guidelines for evaluation. *FAO Food Nutr. Paper* 85 viii–50.

[B27] Gaffen S. Jain R. Garg A. Cua D. (2014). The IL-23-IL-17 immune axis: from mechanisms to therapeutic testing. *Nat. Rev. Immunol.* 14 585–600. 25145755 10.1038/nri3707 PMC4281037

[B28] Gill T. Asquith M. Rosenbaum J. Colbert R. (2015). The intestinal microbiome in spondyloarthritis. *Curr. Opin. Rheumatol.* 27 319–325. 10.1097/BOR.0000000000000187 26002022 PMC4489849

[B29] Guevara-Pacheco S. Feican-Alvarado A. Delgado-Pauta J. Lliguisaca-Segarra A. Pelaez-Ballestas I. (2017). Prevalence of disability in patients with musculoskeletal pain and rheumatic diseases in a population from cuenca, ecuador. *J. Clin. Rheumatol.* 23 324–329. 10.1097/RHU.0000000000000571 28816770

[B30] Guggino G. Mauro D. Rizzo A. Alessandro R. Raimondo S. Bergot A. (2021). Inflammasome activation in ankylosing spondylitis is associated with gut dysbiosis. *Arthritis Rheumatol.* 73 1189–1199. 10.1002/art.41644 33452867

[B31] Guo Z. Zhang J. Wang Z. Ang K. Huang S. Hou Q. (2016). Intestinal microbiota distinguish gout patients from healthy humans. *Sci. Rep.* 6:20602. 10.1038/srep20602 26852926 PMC4757479

[B32] Gurung M. Li Z. You H. Rodrigues R. Jump D. Morgun A. (2020). Role of gut microbiota in type 2 diabetes pathophysiology. *EBioMedicine* 51:102590. 10.1016/j.ebiom.2019.11.051 31901868 PMC6948163

[B33] Häger J. Bang H. Hagen M. Frech M. Träger P. Sokolova M. (2019). The role of dietary fiber in rheumatoid arthritis patients: A feasibility study. *Nutrients* 11:2392. 10.3390/nu11102392 31591345 PMC6836071

[B34] Hashimoto M. Yamazaki T. Hamaguchi M. Morimoto T. Yamori M. Asai K. (2015). Periodontitis and Porphyromonas gingivalis in preclinical stage of arthritis patients. *PLoS One.* 10:e0122121. 10.1371/journal.pone.0122121 25849461 PMC4388350

[B35] He Z. Shao T. Li H. Xie Z. Wen C. (2016). Alterations of the gut microbiome in Chinese patients with systemic lupus erythematosus. *Gut Pathog.* 8:64. 10.1186/s13099-016-0146-9 27980687 PMC5146896

[B36] Heinimann K. von Kempis J. Sauter R. Schiff M. Sokka-Isler T. Schulze-Koops H. (2018). Long-Term increase of radiographic damage and disability in patients with RA in relation to disease duration in the era of biologics. results from the SCQM Cohort. *J Clin Med* 7:57. 10.3390/jcm7030057 29533997 PMC5867583

[B37] Hevia A. Milani C. López P. Cuervo A. Arboleya S. Duranti S. (2014). Intestinal dysbiosis associated with systemic lupus erythematosus. *mBio* 5 e1548–e1514. 10.1128/mBio.01548-14 25271284 PMC4196225

[B38] Hoffmann-Vold A. Fretheim H. Sarna V. Barua I. Carstens M. Distler O. (2021). Safety and efficacy of faecal microbiota transplantation by Anaerobic Cultivated Human Intestinal Microbiome (ACHIM) in patients with systemic sclerosis: study protocol for the randomised controlled phase II ReSScue trial. *BMJ Open.* 11:e048541. 10.1136/bmjopen-2020-048541 34168032 PMC8231046

[B39] Honda K. Littman D. (2016). The microbiota in adaptive immune homeostasis and disease. *Nature* 535 75–84. 10.1038/nature18848 27383982

[B40] Horta-Baas G. Romero-Figueroa M. Montiel-Jarquín A. Pizano-Zárate M. García-Mena J. Ramírez-Durán N. (2017). Intestinal dysbiosis and rheumatoid arthritis: A link between gut microbiota and the pathogenesis of rheumatoid arthritis. *J. Immunol. Res.* 2017:4835189. 10.1155/2017/4835189 28948174 PMC5602494

[B41] Hubbard T. Murray I. Bisson W. Lahoti T. Gowda K. Amin S. (2015). Adaptation of the human aryl hydrocarbon receptor to sense microbiota-derived indoles. *Sci. Rep.* 5:12689. 10.1038/srep12689 26235394 PMC4522678

[B42] Hunter T. Boytsov N. Zhang X. Schroeder K. Michaud K. Araujo A. (2017). Prevalence of rheumatoid arthritis in the United States adult population in healthcare claims databases, 2004–2014. *Rheumatol. Int.* 37 1551–1557. 10.1007/s00296-017-3726-1 28455559

[B43] Johansson L. Sherina N. Kharlamova N. Potempa B. Larsson B. Israelsson L. (2016). Concentration of antibodies against *Porphyromonas gingivalis* is increased before the onset of symptoms of rheumatoid arthritis. *Arthritis Res Ther.* 18:201. 10.1186/s13075-016-1100-4 PMC5015325 27605245

[B44] Jonsson A. Bäckhed F. (2017). Role of gut microbiota in atherosclerosis. *Nat. Rev. Cardiol.* 14 79–87. 10.1038/nrcardio.2016.183 27905479

[B45] Kawar N. Gajendrareddy P. Hart T. Nouneh R. Maniar N. Alrayyes S. (2011). Periodontal disease for the primary care physician. *Dis Mon.* 57 174–183. 21569880 10.1016/j.disamonth.2011.03.003

[B46] Kelly C. Zheng L. Campbell E. Saeedi B. Scholz C. Bayless A. (2015). Crosstalk between microbiota-derived short-chain fatty acids and intestinal epithelial hif augments tissue barrier function. *Cell Host Microbe* 17 662–671. 10.1016/j.chom.2015.03.005 25865369 PMC4433427

[B47] Kharlamova N. Jiang X. Sherina N. Potempa B. Israelsson L. Quirke A. (2016). Antibodies to *Porphyromonas gingivalis* indicate interaction between oral infection, smoking, and risk genes in rheumatoid arthritis etiology. *Arthritis Rheumatol.* 68 604–613. 10.1002/art.39491 26554752 PMC4767537

[B48] Klingberg E. Strid H. Ståhl A. Deminger A. Carlsten H. Öhman L. (2017). A longitudinal study of fecal calprotectin and the development of inflammatory bowel disease in ankylosing spondylitis. *Arthritis Res. Ther.* 19:21. 10.1186/s13075-017-1223-2 28148281 PMC5289027

[B49] Koeth R. Wang Z. Levison B. Buffa J. Org E. Sheehy B. (2013). Intestinal microbiota metabolism of L-carnitine, a nutrient in red meat, promotes atherosclerosis. *Nat. Med.* 19 576–585. 10.1038/nm.3145 23563705 PMC3650111

[B50] Koh A. De Vadder F. Kovatcheva-Datchary P. Bäckhed F. (2016). From dietary fiber to host physiology: Short-Chain fatty acids as key bacterial metabolites. *Cell* 165 1332–1345. 10.1016/j.cell.2016.05.041 27259147

[B51] Kokol P. Blažun Vošner H. Završnik J. (2021). Application of bibliometrics in medicine: a historical bibliometrics analysis. *Health Info Libr. J.* 38 125–138. 10.1111/hir.12295 31995273

[B52] Kragsnaes M. Kjeldsen J. Horn H. Munk H. Pedersen F. Holt H. (2018). Efficacy and safety of faecal microbiota transplantation in patients with psoriatic arthritis: protocol for a 6-month, double-blind, randomised, placebo-controlled trial. *BMJ Open.* 8:e019231. 10.1136/bmjopen-2017-019231 29703851 PMC5922473

[B53] Kumar P. Monin L. Castillo P. Elsegeiny W. Horne W. Eddens T. (2016). Intestinal interleukin-17 receptor signaling mediates reciprocal control of the gut microbiota and autoimmune inflammation. *Immunity* 44 659–671. 10.1016/j.immuni.2016.02.007 26982366 PMC4794750

[B54] Li B. Zhou H. Guo B. Chen W. Tao J. Cao N. (2020). Dysbiosis of oral microbiota is associated with systemic lupus erythematosus. *Arch. Oral Biol.* 113 104708. 10.1016/j.archoralbio.2020.104708 32203722

[B55] Liu X. Zou Q. Zeng B. Fang Y. Wei H. (2013). Analysis of fecal *Lactobacillus* community structure in patients with early rheumatoid arthritis. *Curr. Microbiol.* 67 170–176. 10.1007/s00284-013-0338-1 23483307

[B56] López P. de Paz B. Rodríguez-Carrio J. Hevia A. Sánchez B. Margolles A. (2016). Th17 responses and natural IgM antibodies are related to gut microbiota composition in systemic lupus erythematosus patients. *Sci. Rep.* 6:24072. 10.1038/srep24072 27044888 PMC4820712

[B57] Lucas S. Omata Y. Hofmann J. Böttcher M. Iljazovic A. Sarter K. (2018). Short-chain fatty acids regulate systemic bone mass and protect from pathological bone loss. *Nat. Commun.* 9:55. 10.1038/s41467-017-02490-4 PMC5754356 29302038

[B58] Lundberg K. Kinloch A. Fisher B. Wegner N. Wait R. Charles P. (2008). Antibodies to citrullinated alpha-enolase peptide 1 are specific for rheumatoid arthritis and cross-react with bacterial enolase. *Arthritis Rheum.* 58 3009–3019. 10.1002/art.23936 18821669

[B59] Luo X. Edwards M. Mu Q. Yu Y. Vieson M. Reilly C. (2018). Gut microbiota in human systemic lupus erythematosus and a mouse model of lupus. *Appl. Environ. Microbiol.* 84 e2288–e2217. 10.1128/AEM.02288-17 29196292 PMC5795066

[B60] Luu M. Pautz S. Kohl V. Singh R. Romero R. Lucas S. (2019). The short-chain fatty acid pentanoate suppresses autoimmunity by modulating the metabolic-epigenetic crosstalk in lymphocytes. *Nat. Commun.* 10:760. 10.1038/s41467-019-08711-2 30770822 PMC6377655

[B61] Macfarlane T. Abbood H. Pathan E. Gordon K. Hinz J. Macfarlane G. (2018). Relationship between diet and ankylosing spondylitis: A systematic review. *Eur. J. Rheumatol.* 5 45–52. 10.5152/eurjrheum.2017.16103 29657875 PMC5895151

[B62] Maeda Y. Takeda K. (2017). Role of Gut Microbiota in Rheumatoid Arthritis. *J. Clin. Med.* 6:60. 10.3390/jcm6060060 28598360 PMC5483870

[B63] Maeda Y. Kurakawa T. Umemoto E. Motooka D. Ito Y. Gotoh K. (2016). Dysbiosis contributes to arthritis development via activation of autoreactive T cells in the intestine. *Arthritis Rheumatol.* 68 2646–2661. 10.1002/art.39783 27333153

[B64] Mangat P. Wegner N. Venables P. Potempa J. (2010). Bacterial and human peptidylarginine deiminases: targets for inhibiting the autoimmune response in rheumatoid arthritis? *Arthritis Res. Ther.* 12:209. 10.1186/ar3000 20553633 PMC2911857

[B65] Matcham F. Scott I. Rayner L. Hotopf M. Kingsley G. Norton S. (2014). The impact of rheumatoid arthritis on quality-of-life assessed using the SF-36: a systematic review and meta-analysis. *Semin Arthritis Rheum.* 44 123–130. 10.1016/j.semarthrit.2014.05.001 24973898

[B66] Murray I. Nichols R. Zhang L. Patterson A. Perdew G. (2016). Expression of the aryl hydrocarbon receptor contributes to the establishment of intestinal microbial community structure in mice. *Sci. Rep.* 6:33969. 10.1038/srep33969 27659481 PMC5034278

[B67] Myasoedova E. Crowson C. Kremers H. Therneau T. Gabriel S. (2010). Is the incidence of rheumatoid arthritis rising?: results from Olmsted County, Minnesota, 1955–2007. *Arthritis Rheum.* 62 1576–1582. 10.1002/art.27425 20191579 PMC2929692

[B68] Myers B. Brownstone N. Reddy V. Chan S. Thibodeaux Q. Truong A. (2019). The gut microbiome in psoriasis and psoriatic arthritis. *Best Pract. Res. Clin. Rheumatol.* 33:101494. 10.1016/j.berh.2020.101494 32360228

[B69] Nishida A. Inoue R. Inatomi O. Bamba S. Naito Y. Andoh A. (2018). Gut microbiota in the pathogenesis of inflammatory bowel disease. *Clin. J. Gastroenterol.* 11 1–10. 10.1007/s12328-017-0813-5 29285689

[B70] Okada M. Kobayashi T. Ito S. Yokoyama T. Abe A. Murasawa A. (2013). Periodontal treatment decreases levels of antibodies to *Porphyromonas gingivalis* and citrulline in patients with rheumatoid arthritis and periodontitis. *J. Periodontol.* 84 e74–e84. 10.1902/jop.2013.130079 23701010

[B71] Ortiz P. Bissada N. Palomo L. Han Y. Al-Zahrani M. Panneerselvam A. (2009). Periodontal therapy reduces the severity of active rheumatoid arthritis in patients treated with or without tumor necrosis factor inhibitors. *J. Periodontol.* 80 535–540. 19335072 10.1902/jop.2009.080447 PMC2884010

[B72] Peters V. van de Steeg E. van Bilsen J. Meijerink M. (2019). Mechanisms and immunomodulatory properties of pre- and probiotics. *Benef Microbes.* 10 225–236. 10.3920/BM2018.0066 30827150

[B73] Pianta A. Arvikar S. Strle K. Drouin E. Wang Q. Costello C. (2017). Evidence of the Immune Relevance of Prevotella copri, a Gut Microbe, in Patients With Rheumatoid Arthritis. *Arthritis Rheumatol.* 69 964–975. 10.1002/art.40003 27863183 PMC5406252

[B74] Pritchard A. (1969). Statistical bibliography or bibliometrics. *J. Doc.* 25:348.

[B75] Qin J. Li R. Raes J. Arumugam M. Burgdorf K. Manichanh C. (2010). A human gut microbial gene catalogue established by metagenomic sequencing. *Nature* 464 59–65. 10.1038/nature08821 20203603 PMC3779803

[B76] Quraishi M. Widlak M. Bhala N. Moore D. Price M. Sharma N. (2017). Systematic review with meta-analysis: the efficacy of faecal microbiota transplantation for the treatment of recurrent and refractory Clostridium difficile infection. *Aliment Pharmacol. Ther.* 46 479–493. 28707337 10.1111/apt.14201

[B77] Reid G. Younes J. Van der Mei H. Gloor G. Knight R. Busscher H. (2011). Microbiota restoration: natural and supplemented recovery of human microbial communities. *Nat. Rev. Microbiol.* 9 27–38. 21113182 10.1038/nrmicro2473

[B78] Rosenstein E. Greenwald R. Kushner L. Weissmann G. (2004). Hypothesis: The humoral immune response to oral bacteria provides a stimulus for the development of rheumatoid arthritis. *Inflammation* 28 311–318. 10.1007/s10753-004-6641-z 16245073

[B79] Rossini M. Viapiana O. Vitiello M. Malavolta N. La Montagna G. Maddali Bongi S. (2017). Prevalence and incidence of osteoporotic fractures in patients on long-term glucocorticoid treatment for rheumatic diseases: the Glucocorticoid Induced OsTeoporosis TOol (GIOTTO) study. *Reumatismo* 69 30–39. 10.4081/reumatismo.2017.922 28535619

[B80] Salazar N. Binetti A. Gueimonde M. Alonso A. Garrido P. González del Rey C. (2011). Safety and intestinal microbiota modulation by the exopolysaccharide-producing strains *Bifidobacterium animalis* IPLA R1 and *Bifidobacterium longum* IPLA E44 orally administered to Wistar rats. *Int. J. Food Microbiol.* 144 342–351. 10.1016/j.ijfoodmicro.2010.10.016 21078530

[B81] Scher J. Littman D. Abramson S. (2016). Microbiome in inflammatory arthritis and human rheumatic diseases. *Arthritis Rheumatol.* 68 35–45. 10.1002/art.39259 26331579 PMC4789258

[B82] Scher J. Ubeda C. Artacho A. Attur M. Isaac S. Reddy S. (2015). Decreased bacterial diversity characterizes the altered gut microbiota in patients with psoriatic arthritis, resembling dysbiosis in inflammatory bowel disease. *Arthritis Rheumatol.* 67 128–139. 10.1002/art.38892 25319745 PMC4280348

[B83] Shor D. Dahan S. Comaneshter D. Cohen A. Amital H. (2016). Does inflammatory bowel disease coexist with systemic lupus erythematosus? *Autoimmun Rev.* 15 1034–1037. 27481039 10.1016/j.autrev.2016.07.027

[B84] Simon G. Gorbach S. (1984). Intestinal flora in health and disease. *Gastroenterology.* 86 174–193. 10.1016/0016-5085(84)90606-1 6357937

[B85] Smith P. Howitt M. Panikov N. Michaud M. Gallini C. Bohlooly Y. (2013). The microbial metabolites, short-chain fatty acids, regulate colonic Treg cell homeostasis. *Science* 341 569–573. 10.1126/science.1241165 23828891 PMC3807819

[B86] Snyderman R. McCarty G. (1982). “Analogous mechanisms of tissue destruction in rheumatoid arthritis and periodontal disease,” In *Host-parasite interaction in periodontal diseases*, eds Genco R. Mergenhagen S. Washington, DC: American Society of Microbiology, 354–362.

[B87] Suez J. Korem T. Zeevi D. Zilberman-Schapira G. Thaiss C. Maza O. (2014). Artificial sweeteners induce glucose intolerance by altering the gut microbiota. *Nature.* 514 181–186. 25231862 10.1038/nature13793

[B88] Taurog J. Maika S. Satumtira N. Dorris M. McLean I. Yanagisawa H. (1999). Inflammatory disease in HLA-B27 transgenic rats. *Immunol. Rev.* 169 209–223. 10.1111/j.1600-065X.1999.tb01317.x 10450519

[B89] Toivanen P. (2003). Normal intestinal microbiota in the aetiopathogenesis of rheumatoid arthritis. *Ann. Rheum Dis.* 62 807–811. 10.1136/ard.62.9.807 12922950 PMC1754679

[B90] Tomofuji Y. Maeda Y. Oguro-Igashira E. Kishikawa T. Yamamoto K. Sonehara K. (2021). Metagenome-wide association study revealed disease-specific landscape of the gut microbiome of systemic lupus erythematosus in Japanese. *Ann. Rheum. Dis.* 80 1575–1583. 10.1136/annrheumdis-2021-220687 34426398 PMC8600607

[B91] Torres-Fuentes C. Schellekens H. Dinan T. Cryan J. (2017). The microbiota-gut-brain axis in obesity. *Lancet Gastroenterol. Hepatol.* 2 747–756. 28844808 10.1016/S2468-1253(17)30147-4

[B92] Van de Wiele T. Van Praet J. Marzorati M. Drennan M. Elewaut D. (2016). How the microbiota shapes rheumatic diseases. *Nat. Rev. Rheumatol.* 12 398–411. 10.1038/nrrheum.2016.85 27305853

[B93] van Eck N. Waltman L. (2010). Software survey: VOSviewer, a computer program for bibliometric mapping. *Scientometrics* 84 523–538. 20585380 10.1007/s11192-009-0146-3 PMC2883932

[B94] Volkmann E. (2017). Intestinal microbiome in scleroderma: recent progress. *Curr Opin Rheumatol.* 29 553–560. 28719392 10.1097/BOR.0000000000000429

[B95] Volkmann E. Chang Y. Barroso N. Furst D. Clements P. Gorn A. (2016). Association of systemic sclerosis with a unique colonic microbial consortium. *Arthritis Rheumatol.* 68 1483–1492. 10.1002/art.39572 26749064 PMC5561666

[B96] Wade W. (2013). The oral microbiome in health and disease. *Pharmacol. Res.* 69 137–143. 10.1016/j.phrs.2012.11.006 23201354

[B97] Wang W. Zhou H. Liu L. (2018). Side effects of methotrexate therapy for rheumatoid arthritis: A systematic review. *Eur. J. Med. Chem.* 158 502–516. 10.1016/j.ejmech.2018.09.027 30243154

[B98] Wegner N. Lundberg K. Kinloch A. Fisher B. Malmström V. Feldmann M. (2010). Autoimmunity to specific citrullinated proteins gives the first clues to the etiology of rheumatoid arthritis. *Immunol. Rev.* 233 34–54. 10.1111/j.0105-2896.2009.00850.x 20192991

[B99] Wen C. Zheng Z. Shao T. Liu L. Xie Z. Le Chatelier E. (2017). Quantitative metagenomics reveals unique gut microbiome biomarkers in ankylosing spondylitis. *Genome Biol.* 18:142. 10.1186/s13059-017-1271-6 PMC5530561 28750650

[B100] Whitmore S. Lamont R. (2014). Oral bacteria and cancer. *PLoS Pathog.* 10:e1003933. 10.1371/journal.ppat.1003933 24676390 PMC3968118

[B101] Xiang L. Low A. Leung Y. Fong W. Gan W. Graves N. (2020). Work disability in rheumatic diseases: Baseline results from an inception cohort. *Int J Rheum Dis.* 23 1040–1049. 32512639 10.1111/1756-185X.13864

[B102] Ye Z. Zhang N. Wu C. Zhang X. Wang Q. Huang X. (2018). A metagenomic study of the gut microbiome in Behcet’s disease. *Microbiome* 6:135. 10.1186/s40168-018-0520-6 PMC6091101 30077182

[B103] Yeoh N. Burton J. Suppiah P. Reid G. Stebbings S. (2013). The role of the microbiome in rheumatic diseases. *Curr. Rheumatol. Rep.* 15:314. 10.1007/s11926-012-0314-y 23378145

[B104] Zaiss M. Jones R. Schett G. Pacifici R. (2019). The gut-bone axis: how bacterial metabolites bridge the distance. *J. Clin. Invest.* 129 3018–3028. 10.1172/JCI128521 31305265 PMC6668676

[B105] Zamani B. Golkar H. Farshbaf S. Emadi-Baygi M. Tajabadi-Ebrahimi M. Jafari P. (2016). Clinical and metabolic response to probiotic supplementation in patients with rheumatoid arthritis: a randomized, double-blind, placebo-controlled trial. *Int. J. Rheum Dis.* 19 869–879. 27135916 10.1111/1756-185X.12888

[B106] Zeng H. Chi H. (2015). Metabolic control of regulatory T cell development and function. *Trends Immunol.* 36 3–12. 10.1016/j.it.2014.08.003 25248463 PMC4280284

[B107] Zhang X. Zhang D. Jia H. Feng Q. Wang D. Liang D. (2015). The oral and gut microbiomes are perturbed in rheumatoid arthritis and partly normalized after treatment. *Nat. Med.* 21 895–905. 10.1038/nm.3914 26214836

