# Supplementary material for: Effects of intestinal microbes on rheumatic diseases: A bibliometric analysis
Source: Front Microbiol. 2023 Jan 9;13:1074003. doi: 10.3389/fmicb.2022.1074003 (PMC9870327; doi:10.3389/fmicb.2022.1074003)
Supplement: Supplementary file 1 [file Data_Sheet_1.docx]

**Supplementary Materials**

**
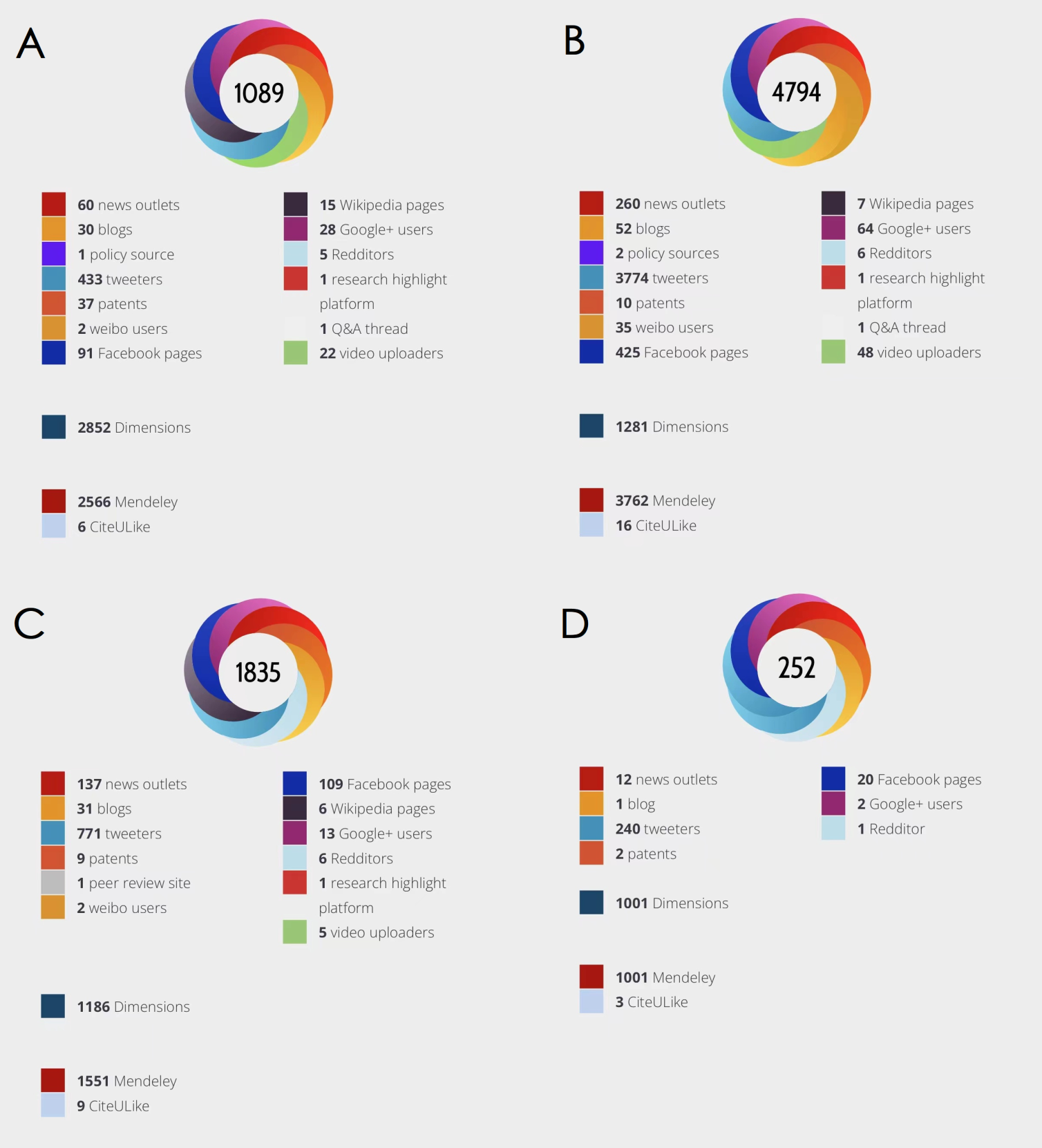
**

**Figure S1.** Altmetric Attention Score (AAS). **(A)** AAS of ‘Intestinal microbiota metabolism of l-carnitine, a nutrient in red meat, promotes atherosclerosis’, published in Nature Medicine, April 2013. **(B)** AAS of ‘Artificial sweeteners induce glucose intolerance by altering the gut microbiota, published in Nature’, September 2014. **(C)** AAS of ‘Dietary emulsifiers impact the mouse gut microbiota promoting colitis and metabolic syndrome’, published in Nature, February 2015. **(D)** AAS of ‘The oral and gut microbiomes are perturbed in rheumatoid arthritis and partly normalized after treatment’, published in Nature Medicine, July 2015.

**
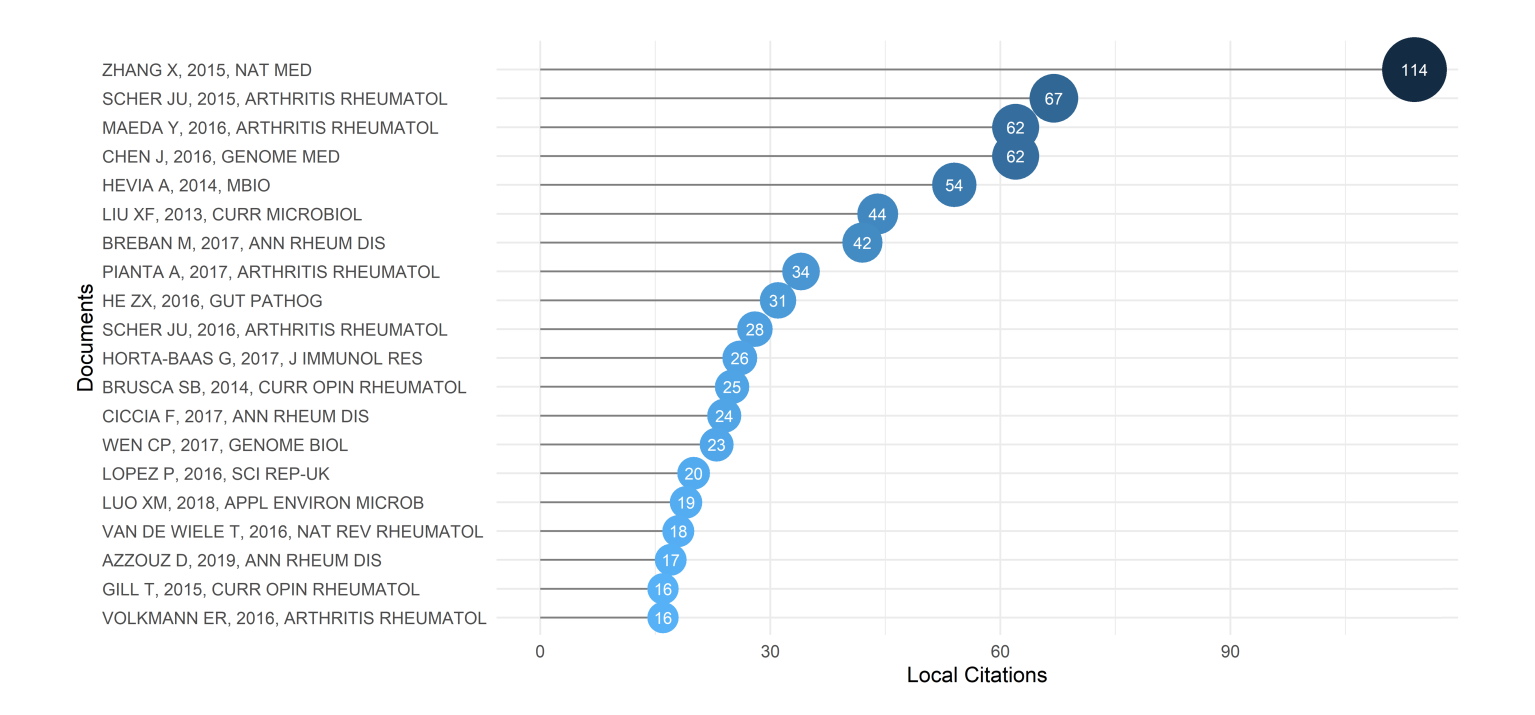
**

**Figure S2.** Most local cited documents. Local cited documents refer to the documents cited by other articles in our database.

**
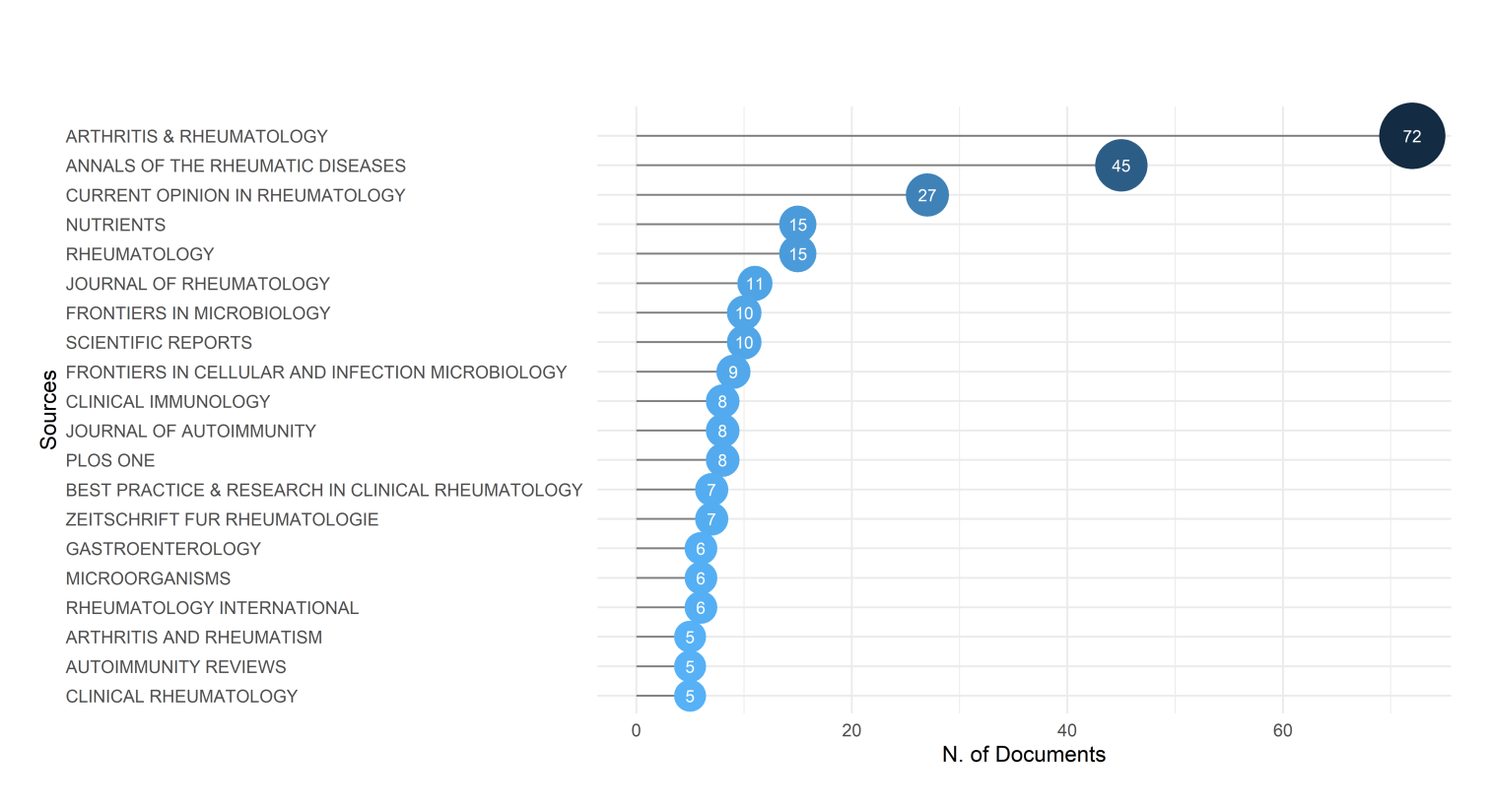
**

**Figure S3.** Most relevant sources.

**
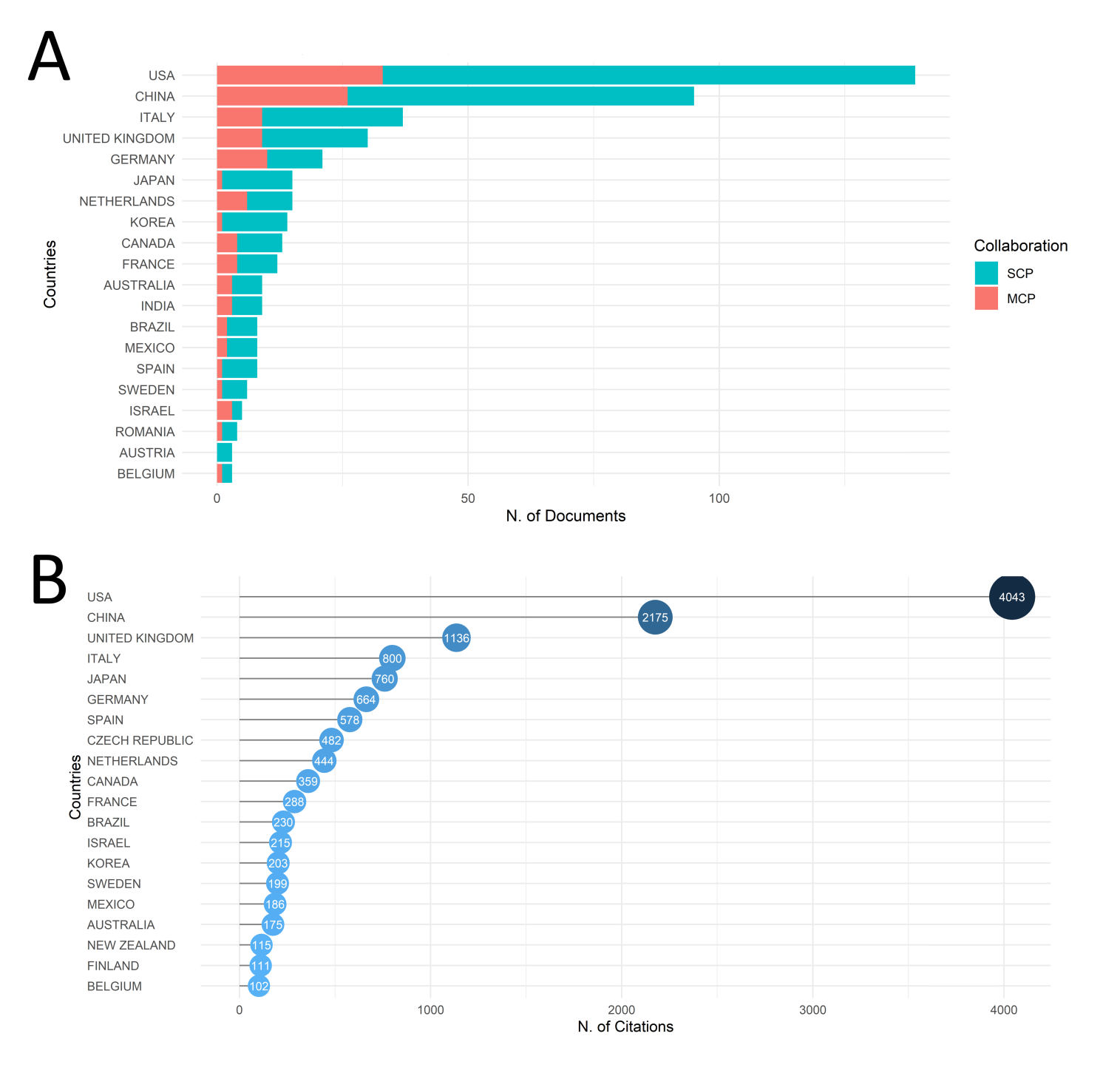
**

**Figure S4. (A)** Corresponding author’s country. **(B)** Most cited countries. SCP: Single country publications, MCP: Multiple country publications.

**Table S1.** Country production.

| **Region** | **Frequency** |
| --- | --- |
| USA | 527 |
| CHINA | 330 |
| ITALY | 191 |
| UK | 116 |
| JAPAN | 111 |
| GERMANY | 101 |
| FRANCE | 88 |
| SPAIN | 78 |
| AUSTRALIA | 56 |
| NETHERLANDS | 55 |
| CANADA | 48 |
| SOUTH KOREA | 42 |
| MEXICO | 41 |
| NORWAY | 34 |
| SWEDEN | 34 |
| BRAZIL | 30 |
| INDIA | 28 |
| BELGIUM | 23 |
| PORTUGAL | 20 |
| ROMANIA | 20 |

Frequency refers to the number of documents of the country.
