# Supplementary material for: Effects of intestinal microbes on rheumatic diseases: A bibliometric analysis
Source: Front Microbiol. 2023 Jan 9;13:1074003. doi: 10.3389/fmicb.2022.1074003 (PMC9870327; doi:10.3389/fmicb.2022.1074003)
Supplement: Supplementary file 2 [file Table_2.docx]

|  | **Pathology** | | **Disease** | **Treatment** | **Experiments** |
| --- | --- | --- | --- | --- | --- |
|  | **Macro level** | **Micro level** |  |  |  |
| Cluster 1 (purple):  Intestinal bacteria lead to rheumatic diseases through joints’ inflammation. | inflammation, pathogenesis, association, activation, intestinal permeability |  | disease | probiotic supplementation, therapy, supplementation, lactobacillus casei | model, mice, susceptibility |
| Cluster 2 (blue):  Rheumatic diseases are closely associated with inflammatory bowel disease. | fecal microbiota, gut inflammation, intestinal inflammation, intestinal microbiome | t-cells, unfolded protein response, faecalibacterium-prausnitzii, innate lymphoid cells, Th17 cells, dendritic cells | ankylosing spondylitis, inflammatory bowel disease, psoriatic arthritis, Crohn’s disease, ulcerative colitis, systemic lupus erythematosus, enthesitis related arthritis, early rheumatoid arthritis, reactive arthritis, axial spondyloarthritis, bowel disease |  | genome-wide association, transgenic rats, synovial fluid, peripheral blood, |
| Cluster 3 (red):  Rheumatic diseases and intestinal microbiota are interrelated by immunoregulation. | gut microbiota, fecal microbiota, metabolism, immune responses, mechanisms, immune system | chain fatty acids, regulatory t-cells, segmented filamentous bacteria, aryl hydrocarbon receptor, molecular mimicry, methotrexate | rheumatoid arthritis, obesity, knee osteoarthritis, autoimmune arthritis, | nonsteroidal anti-inflammatory drugs | Bacterial DNA, sequences, hla b27 |
| Cluster 4 (orange):  Intestinal dysbiosis and loss of microbial diversity exist in patients with rheumatic diseases. | dysbiosis, diversity, cells, microbiota, infection, prevalence, immunity, epidemiology | macrophages | spondyloarthritis, colitis, collagen-induced arthritis |  | double-blind |
| Cluster 5 (green):  The occurrence of rheumatic diseases is also related to the oral flora. | autoimmunity, induction | porphyromonas gingivalis, antibodies, autoantibodies, shared epitope | periodontal disease |  |  |

**TableS2.** Taxonomy of themes and research dimensions categories.
